# Mesopic Functional Visual Acuity in Normal Subjects

**DOI:** 10.1371/journal.pone.0134505

**Published:** 2015-07-28

**Authors:** Takahiro Hiraoka, Sujin Hoshi, Yoshifumi Okamoto, Fumiki Okamoto, Tetsuro Oshika

**Affiliations:** Department of Ophthalmology, Faculty of Medicine, University of Tsukuba, Ibaraki, Japan; University of Western Australia, AUSTRALIA

## Abstract

To evaluate mesopic functional visual acuity (FVA) with a newly developed system in normal subjects and to compare the results with photopic FVA, sixty-eight healthy volunteers (24.03 ± 4.42 [mean ± standard deviation] years) were enrolled in this study. A commercially available FVA measurement system (AS-28; Kowa, Aichi, Japan) was modified to measure FVA under mesopic conditions as well as photopic conditions. Measurements were performed monocularly in photopic conditions during 60 seconds. After dark adaptation for 15 minutes, the same measurements were repeated in mesopic conditions. Outcomes included starting visual acuity (VA), FVA (the average of VAs), visual maintenance ratio (VMR), maximum VA, minimum VA, and numbers of blinks during the 60-second measurement session, and were compared between mesopic and photopic conditions. Starting VA was –0.11 ± 0.08 and 0.39 ± 0.12 logarithm of the minimum angle of resolution (logMAR) in photopic and mesopic conditions, respectively. FVA was –0.06 ± 0.09 and 0.52 ± 0.14 logMAR, VMR was 0.98 ± 0.02 and 0.94 ± 0.04, maximum VA was –0.15 ± 0.06 and 0.33 ± 0.12 logMAR, the minimum VA was 0.05 ± 0.12 and 0.78 ± 0.20 logMAR, and the number of blinks was 8.23 ± 7.54 and 7.23 ± 6.20, respectively. All these parameters except the number of blinks were significantly different between the two conditions (*P* < 0.001). Besides, the difference between maximum and minimum VAs and standard deviation of VA were significantly larger in mesopic than in photopic conditions (*P* < 0.001). This study revealed that not only overall visual function decline but also instability of vision under mesopic conditions even in healthy subjects.

## Introduction

Standard visual acuity (VA), which relies on a patient’s recognition of familiar, high contrast letters or Landolt rings, is an excellent measure of visual function. However, visual acuity is only one aspect of actual visual function. Contrast sensitivity and glare testing provide additional important details of visual function. Unfortunately, commonly used tests to measure these aspects of visual function only detect the maximum value of each parameter. Therefore, results are expressed as one value and may not accurately reflect the entire spectrum of results. In many situations of daily life, continuous gazing tasks (e.g., reading, driving, and visual display terminal work) are required and patients often complain of visual disturbances even though they have excellent standard VA.

In recent years, sequential changes in visual function have received much attention. This is because everyday tasks generally involve continuous, not static, acquisition of visual information. Functional visual acuity (FVA) testing has been developed to assess dynamic changes in visual function under photopic conditions [1–4]. These testing results are thought to be important indicators of visual performance related to daily activities [5]. The FVA measurement was initially used to detect visual function impairment in dye eye patients with normal standard VA who complained of decreased visual function [1,2,5,6]. Kaido et al. [7] have suggested that FVA measurements can be used as a screening tool for dry eye syndrome. The FVA testing has also been shown to be useful in assessing dynamic VA changes in eyes with mild cataract opacities [8], posterior capsule opacification after cataract surgery [9], Stevens-Johnson syndrome [3], and laser in situ keratomileusis [10]. Furthermore, slight deteriorations in vision caused by the use of viscous eyedrops [11], eye ointment [12], and contact lens wear [13] were successfully detected with the FVA system. The FVA testing even detected early changes in central visual function due to retinal disease, such as epiretinal membrane, which were not detected with standard VA testing [14].

Under low lighting conditions, including night time and overcast weather conditions with rain or fog, standard VA is less important to overall visual function than the ability to discriminate dim contrasts [15]. Patients who have undergone corneal refractive surgery frequently complain of night vision disturbances and glare, even though high-contrast VA is excellent [16–22]. Thus, visual function in low luminance (mesopic) conditions should be examined along with visual function in high luminance (photopic) conditions.

Thus far, numerous studies have evaluated the relationship between visual performance and traffic accidents. There have been several studies that showed the positive associations between visual acuity and crash involvement, although the degree of each association is not so high [23–25]. On the other hand, some reports denote the negative relationship between visual acuity and traffic accidents [26–28]. On the whole, there is little support for a strong association between visual acuity and unsafe driving [29]. More recently, Subzwari et al. [30] have also noted that there is insufficient evidence to show the effect of vision screening tests on subsequent motor vehicle crash reduction, and there is a need to develop valid and reliable tools of vision screening that can predict driving performance.

The rate of fatal accidents (number of accidents per mile driven) has been reported to be three to four times higher at night than during the day [21,22,31,32]. In addition, crash severity is at least two times higher at night than during the day [33–35]. Unfortunately, conventional vision tests are not useful in predicting driving performance or accident rates [29]. These findings evidently show that studies of visual performance under low lighting conditions are of considerable importance for driving. Given that traffic volume is increasing around the world and that a significant proportion of traffic accidents occur at night [21], it is crucial to examine dynamic visual function under mesopic conditions. In the current study, we examined and compared dynamic VA in normal subjects under both mesopic and photopic lighting conditions using the FVA measurement system.

## Subjects and Methods

### Participants

Healthy volunteers with a best-corrected visual acuity (BCVA) of 20/20 or better were enrolled in this study. Subjects were excluded if they had any systemic or ocular disease (except for refractive error), prior eye surgery or trauma, or dry eye symptoms. Subjects who regularly used eye drops or contact lenses were also excluded. The study protocol was approved by the University of Tsukuba Hospital Institutional Review Board and all study conduct adhered to the tenets of the Declaration of Helsinki. After the study protocol had been fully explained, all subjects provided written informed consent to participate in the study.

### Functional visual acuity measurement system

The FVA measurement system (AS-28; Kowa, Aichi, Japan) was used to examine dynamic changes in VA over time. This system has been described in detail elsewhere [1–4]. Briefly, Landolt optotypes were presented inside the device, beginning at the size equivalent of the subject’s baseline BCVA. The test was performed under photopic conditions on one eye at a time while the subject wore spectacles containing the refractive correction needed to obtain BCVA. The device had a background luminance of 250 ± 25 cd/m^2^. Subjects delineate automatically presented Landolt ring orientation by manipulating the joystick. Optotype size changed by one step, depending on subject response. If the response was correct, a smaller optotype was presented as the next stimulus; if the response was incorrect, a larger optotype was presented as the next stimulus. When the subject did not respond within 2 seconds, the stimulus was assumed to be incorrectly identified. Testing was continuously performed for 60 seconds while the subject was allowed to blink naturally.

**Fig 1:**
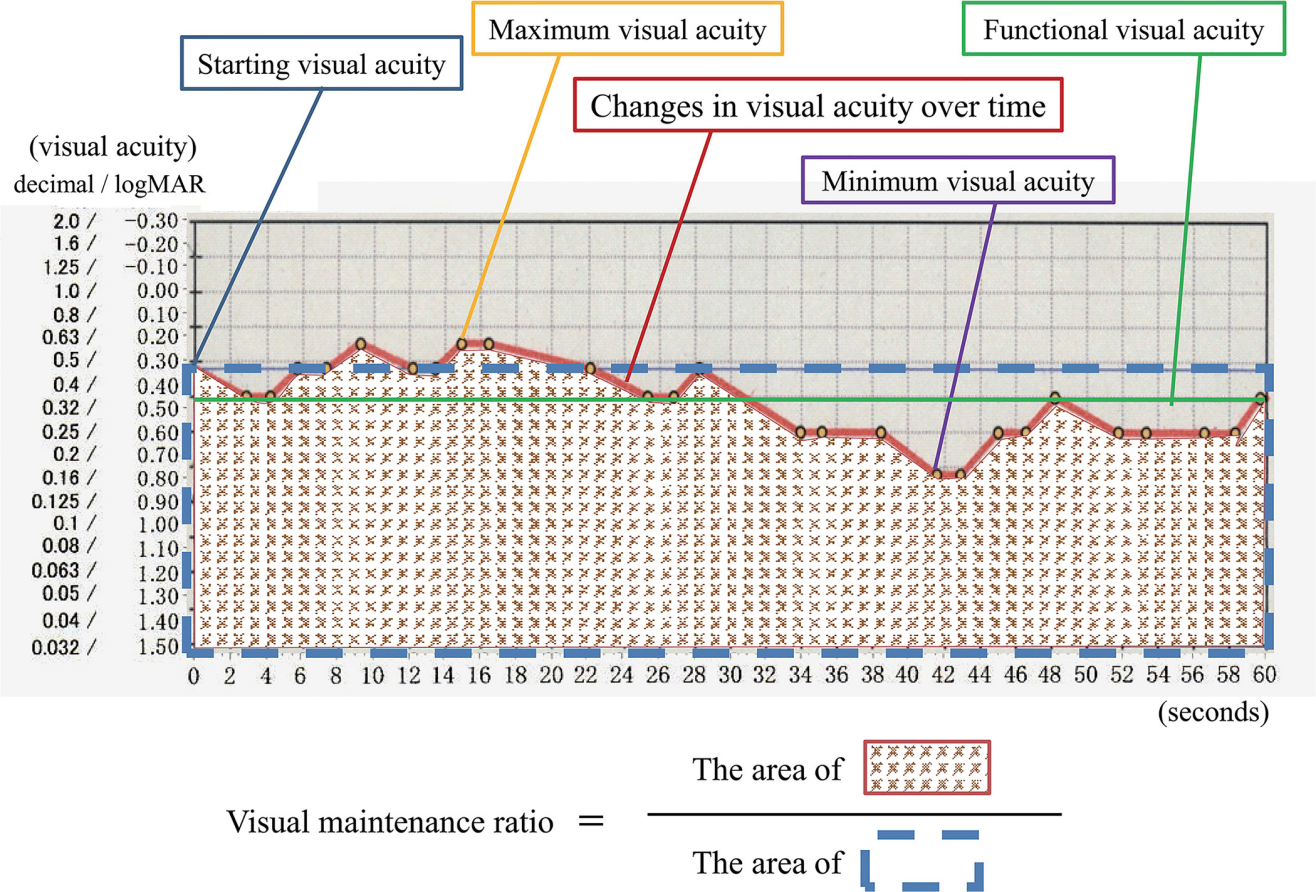
Various parameters of functional visual acuity testing. Sequential visual acuities measured over a 60-second measurement session are denoted by continuous red line. Yellow dots show correct responses. Average of all visual acuity values is calculated as functional visual acuity, which is shown as green line. Blue line denotes starting visual acuity. Visual maintenance ratio refers to area beneath time-wise change in visual acuity (red line) divided by area beneath starting visual acuity (blue line). Maximum and minimum visual acuities imply the best and worst value of visual acuity over the testing period.

When each FVA test was complete, results were recorded as a line graph made up of points joining only the correct answers. Testing outcome parameters included starting VA, FVA, visual maintenance ratio (VMR), maximum VA, minimum VA, and number of blinks. The starting VA was defined as the standard BCVA just before the 60 second measurement session. The FVA was defined as the mean value of time-dependent VA changes during the overall examination. The VMR was defined as the FVA divided by the baseline VA [3]. This index allows different patient groups to be statistically compared, even when groups have different baseline VAs [3–4]. Maximum VA and minimum VA were defined as the best VA and the worst VA recorded during the examination, respectively (Fig 1) [36].

The original FVA measurement system only had a photopic background luminance, but we improved the internal constitution so that another background luminance region could be selected. Mesopic background luminance was set at 0.1 ± 0.01 cd/m^2^ behind the dial of optotypes. The two luminance levels could be easily switched.

### Measuring sequence

First of all, we performed the FVA measurements in photopic conditions. After completing photopic FVA measurements, subjects were dark-adapted by having them rest in a dark room for 15 minutes. The background luminance of the system was switched to the mesopic range and the FVA measurements were repeated. To adjust for night myopia, an additional minus lens between 0.00 and 1.00 diopters (D) in strength, was added to the subject’s spectacle correction.

### Statistical methods

Only data from the right eye of each subject was included in this study. Where applicable, data are presented as mean ± standard deviation. Outcomes were compared between mesopic and photopic conditions using Wilcoxon’s signed rank test, because the obtained data were not normally distributed. Statistical significance was defined as a *P* < 0.05. All statistical analyses were performed using SPSS version 15.0J software (SPSS Inc., Chicago, IL).

## Results

A total of 68 healthy volunteers (40 males, 28 females) were included in this study. Mean subject age was 24.03 ± 4.42 years (range: 21 to 43 years) and the spherical equivalent of the refractive error was -2.97 ± 2.87 D (range: -11.50 to 0.00 D). Average baseline BCVA was -0.14 ± 0.07 (range: -0.30 to 0.00) logarithm of the minimum angle of resolution (logMAR).

Under photopic conditions, starting VA was -0.11 ± 0.08 logMAR, FVA was -0.06 ± 0.09 logMAR, VMR was 0.98 ± 0.02, maximum VA was -0.15 ± 0.06 logMAR, minimum VA was 0.05 ± 0.12 logMAR, and number of blinks was 8.23 ± 7.54. The average difference between maximum VA and minimum VA was 0.20 ± 0.09 and the standard deviation of VA measured over the 60 second testing period was 0.02 ± 0.01.

Under mesopic conditions, starting VA was 0.39 ± 0.12 logMAR, FVA was 0.52 ± 0.14 logMAR, VMR was 0.94 ± 0.04, maximum VA was 0.33 ± 0.12 logMAR, minimum VA was 0.78 ± 0.20 logMAR, and number of blinks was 7.23 ± 6.20. The difference between maximum and minimum VA was 0.45 ± 0.16 and the standard deviation of VA measured over the 60 second testing period was 0.04 ± 0.02. All parameters, with the exception of the number of blinks, were significantly different between photopic and mesopic conditions (*P* < 0.001, Table 1).

**Table 1:** Comparison of visual function parameters between photopic and mesopic conditions.

	Photopic condition (mean ± SD)	Mesopic condition (mean ± SD)	*P* value
Starting VA (logMAR)	-0.11 ± 0.08	0.39 ± 0.12	*P* < 0.001*
Functional VA (logMAR)	-0.06 ± 0.09	0.52 ± 0.14	*P* < 0.001*
Visual maintenance ratio	0.98 ± 0.02	0.94 ± 0.04	*P* < 0.001*
Maximum VA (logMAR)	-0.15 ± 0.06	0.33 ± 0.12	*P* < 0.001*
Minimum VA (logMAR)	0.05 ± 0.12	0.78 ± 0.20	*P* < 0.001*
Number of blinks	8.23 ± 7.54	7.23 ± 6.20	*P* = 0.608
Difference between maximum VA and minimum VA	0.204 ± 0.090	0.446 ± 0.159	*P* < 0.001*
Standard deviation of VA during 60 seconds	0.019 ± 0.013	0.040 ± 0.022	*P* < 0.001*

VA = visual acuity, logMAR = logarithm of the minimum angle of resolution, SD = standard deviation.

*: Significant difference by Wilcoxon’s signed rank test.

**Fig 2:**
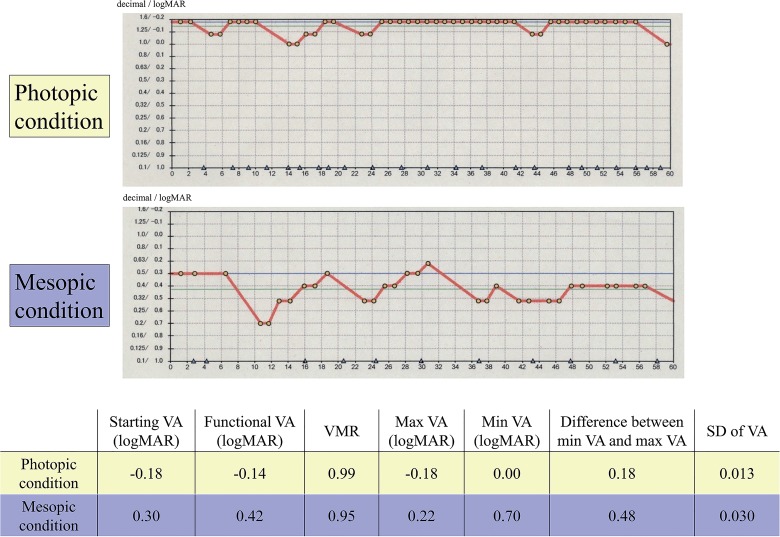
Results of functional visual acuity testing in both photopic and mesopic conditions in a representative subject (22 year old female). All visual outcomes in mesopic conditions were apparently worse than those in photopic conditions. In addition, it should be noted that line graph of visual acuity during 60 seconds remarkably fluctuated in mesopic conditions. VA = visual acuity, max = maximum, min = minimum, SD = standard deviation, logMAR = logarithm of the minimum angle of resolution.

Fig 2 shows representative FVA testing results. When results between the two background lighting conditions were compared, all visual outcomes measured under mesopic conditions were apparently worse than those under photopic conditions. It should also be noted that the line graph presenting VA changes over the 60 second testing period showed remarkable VA fluctuations under mesopic conditions.

The effect of subject age and refractive error was examined for each visual parameter measured. No significant correlations were identified (R = -0.221–0.153, P = 0.070–0.983, Spearman’s rank correlation coefficient).

## Discussion

We developed and tested a new method to measure mesopic FVA using a commercially available FVA measurement system. The measurements were performed after dark adaptation for 15 minutes, because numerous studies have evaluated mesopic visual functions after dark adaptation for 5–20 minutes [37–39] and there has been a report which showed better and more stable results after 15 minutes of adaptation compared to 5 minutes in some subjects [40]. It has been shown that a myopic shift can occur in low luminance conditions (i.e., “night myopia”). This shift is typically between 0.50 and 1.00 D in young adults, although it varies considerably between subjects [41,42]. Several researchers have suggested that optical correction of night myopia would be of only marginal benefit for visual acuity [43,44]. However, we did correct each subject’s myopic shift after dark adaptation (-2.97 ± 2.87 D under photopic conditions, -3.34 ± 2.93 D under mesopic conditions) so that suboptimal refractive correction and intersubject myopic shift variation would not confound our results.

In this study, we demonstrated that visual function measures all deteriorated in mesopic conditions compared to photopic conditions. Our results are in agreement with the study by Johnson [44], who measured VA under several different low luminance levels using sinusoidal gratings. Even with night myopia correction, VA was significantly decreased in mesopic conditions compared with photopic conditions. This result strongly suggests that factors other than refraction influence visual acuity under low luminance conditions. It has been suggested that decreases in VA occur at lower light levels because of neural factors associated with decreasing retinal illuminance, and not optical blur from myopic shifts [43,44]. Our results further support this idea.

It is understandable that not only starting VA but also maximum VA and minimum VA worsened under mesopic conditions in our study, because a loss in VA with a decrease in luminance has been previously reported [43,44]. Additionally, because FVA is the average of all VA measurements obtained during the testing period, it was not surprising that FVA also declined in mesopic conditions. The FVA measurement is thought to reflect daily vision more effectively than single VA measurements at one point in time [4]. Therefore, our results show that visual function generally decreases under mesopic conditions.

Interestingly, line graphs of VA during the 60 second testing period markedly fluctuated and VMR was significantly lower in mesopic conditions than in photopic conditions. We also found that visual function was more unstable over the 60 second testing period during mesopic conditions, as indicated by a larger VA range and a greater VA standard deviation during mesopic conditions than during photopic conditions. These findings imply that it is difficult for the human eye to maintain a constant VA in dark circumstances. Although our data cannot help explain why this occurs, we infer that microfluctuations in accommodation contribute to VA instability. The results of several studies support this theory, demonstrating that accommodation does vary under low luminance conditions [45–48]. Collins [49] first noted the presence of small amplitude temporal variations in accommodation response while viewing a stationary object. Campbell et al. [50,51] successfully used Fourier analysis to identify two dominant frequency components within the waveform, which they termed microfluctuations; a low frequency component (LFC < 0.6 Hz) and a high frequency component (1.0 < HFC < 2.3 Hz) [47,50–52]. The HFC represents physiological noise within the accommodation system, which is correlated with arterial pulse, but directional information can be provided by the LFC [48,53]. A fluctuation in one direction tends to bring an out-of-focus image into focus, while a fluctuation in the other direction does the opposite. Thus, fluctuations could provide an odd-error cue to the visual system to allow optimal retinal image quality to be achieved [45,54]. Gray et al. [48] suggested that LFC power increases that occur with target luminance decreases represent accommodation system attempts to maintain a constant feedback of retinal image quality under degraded stimulus conditions [48]. In other words, if the accommodation control system is not receiving sufficient information on retinal image quality, it would cause greater microfluctuations in an effort to maintain the required level of variation in retinal image quality. Based on these findings, the VA fluctuations during mesopic conditions that were observed in our study may be explained by accommodation variability at low luminance levels. Another possible explanation for the VA fluctuations is that eye movement variability increases in the dark [55]. Given the variability of photoreceptor density in the central retina, even minor changes in eye movement and fixation stability likely result in changes in visual acuity. There is one more possible explanation associated with neural factors. Plainis and Murray [35,56] examined reaction times to targets (sinusoidal gratings) presented in a wide range of luminance which are influenced by anatomical and physiological characteristics of the retino-cortical pathway, and showed that the reaction times increase as luminance is decreased. That is, visual central processing times to recognize objects become longer in mesopic and scotopic conditions than in photopic conditions. Thus, the prolonged processing time may induce VA instability in our study, because our test needs continuous and relatively quick responses to optotypes presented. Further studies are needed to confirm these hypotheses.

To the best of our knowledge, this is the first study to examine dynamic VA changes under mesopic conditions in normal subjects. Driving at night or in bad conditions (e.g., heavy rain or fog) requires optimal continuous vision and our finding that considerable VA fluctuations occur during mesopic conditions in healthy eyes is quite important. It needs to be informed not only ophthalmologists and patients but also the whole society so that the number of life-threatening traffic accidents at night and in bad weather conditions can be reduced. Similar dynamic VA measurements should also be made on elderly subjects to investigate mesopic visual function stability in this population, because elderly drivers are known to be more susceptible to VA loss during low lighting conditions [57].

Our study did have some limitations. Because accommodation and eye movement were not evaluated in the current study, the mechanism underlying mesopic VA fluctuation has been speculated upon, but remains unknown. Future studies specifically designed to understand the role of accommodation and eye movement on mesopic visual acuity maintenance should be performed. Another study weakness was that optical quality parameters (e.g., higher-order wavefront aberrations and light scattering) were not examined. Changes in optical quality may also contribute to visual function degradation and instability under mesopic conditions and should be investigated. Furthermore, our study population only included subjects within a narrow age range. This meant that age-related changes in FVA could not be evaluated. As discussed above, studies including subjects of a wider age range should be conducted. Finally, the relationship between mesopic vision instability and night driving accidents must be investigated in a well-designed study.

In conclusion, this was the first attempt to assess dynamic VA changes under mesopic conditions. Using our newly developed system on normal subjects, we revealed not only overall visual function decline but also instability of vision under mesopic conditions. Educating the public on mesopic vision instability may help to reduce the number of road accidents. Additionally, our results warrant further investigation on mesopic functional vision in the elderly and in patients with common ocular pathologies, including corneal opacity, cataract, glaucoma, and vitreoretinal disease. We suppose that our mesopic FVA methods are quite sensitive to early, tiny changes in visual function. This testing is applicable to clinical practice and may help physicians identify and understand unknown visual complaints especially under dark conditions in patients with good standard VA.

## Supporting Information

S1 Dataset Detailed information for all subjects. (XLSX)
